# 12-month mortality and loss-to-program in antiretroviral-treated children: The IeDEA pediatric West African Database to evaluate AIDS (pWADA), 2000-2008

**DOI:** 10.1186/1471-2458-11-519

**Published:** 2011-06-30

**Authors:** Didier K Ekouevi, Alain Azondekon, Fatoumata Dicko, Karen Malateste, Pety Touré, François T Eboua, Kouakou Kouadio, Lorna Renner, Kevin Peterson, François Dabis, Haby Signaté Sy, Valeriane Leroy

**Affiliations:** 1 INSERM, U897, & Institut de Santé Publique, Epidémiologie et Développement (ISPED), Université Victor Segalen Bordeaux 2, Bordeaux, France; 2 Programme PAC-CI, CHU de Treichville, Abidjan, Côte d'Ivoire; 3 Hôpital d'Instruction des Armées, Cotonou, Benin; 4 Hôpital Gabriel Touré, Service de Pédiatrie Bamako, Mali; 5 MTCT-Plus initiative, ACONDA, Abidjan, Côte d'Ivoire; 6 CePReF, ACONDA, Abidjan Côte d'Ivoire; 7 CHU de Yopougon, Service de Pédiatrie, Abidjan, Côte d'Ivoire; 8 Centre Intégré de Recherche Bioclinique d'Abidjan (CIRBA), Abidjan, Côte d'Ivoire; 9 Korlebu Hospital, Pediatric ward, Accra, Ghana; 10 Medical Research Council, Fajara Cohort, Banjul, Gambia; 11 Hôpital d'Enfants Albert-Royer, Dakar, Senegal

## Abstract

**Background:**

The IeDEA West Africa Pediatric Working Group (pWADA) was established in January 2007 to study the care and treatment of HIV-infected children in this region. We describe here the characteristics at antiretroviral treatment (ART) initiation and study the 12-month mortality and loss-to-program of HIV-infected children followed in ART programs in West Africa.

**Methods:**

Standardized data from HIV-infected children followed-up in ART programs were included. Nine clinical centers from six countries contributed to the dataset (Benin, Côte d'Ivoire, Gambia, Ghana, Mali and Senegal). Inclusion criteria were the followings: age 0-15 years and initiated triple antiretroviral drug regimens. Baseline time was the date of ART initiation. WHO criteria was used to define severe immunosuppression based on CD4 count by age or CD4 percent < 15%. We estimated the 12-month Kaplan-Meier probabilities of mortality and loss-to-program (death or loss to follow-up > 6 months) after ART initiation and factors associated with these two outcomes.

**Results:**

Between June 2000 and December 2007, 2170 children were included. Characteristics at ART initiation were the following: median age of 5 years (Interquartile range (IQR: 2-9) and median CD4 percentage of 13% (IQR: 7-19). The most frequent drug regimen consisted of two nucleoside reverse transcriptase inhibitors and one non-nucleoside reverse transcriptase inhibitors (62%). During the first 12 months, 169 (7.8%) children died and 461(21.2%) were lost-to-program. Overall, in HIV-infected children on ART, the 12-month probability of death was 8.3% (95% Confidence Interval (CI): 7.2-9.6%), and of loss-to-program was 23.1% (95% CI: 21.3-25.0%). Both mortality and loss-to program were associated with advanced clinical stage, CD4 percentage < 15% at ART initiation and year (> 2005) of ART initiation.

**Conclusion:**

Innovative and sustainable approaches are needed to better document causes of death and increase retention in HIV pediatric clinics in West Africa.

## Background

Pediatric HIV infection is a worldwide public health challenge that disproportionately affects children in the poorest parts of the world. By December 2008, an estimated 33.4 million people were living with HIV, 2.1 million of whom were children under 15 years of age [1]. Furthermore, 16% of the 2.7 million new HIV infections in 2008 occurred in children [1]. At the end of 2009, 356,400 HIV-infected children younger than 15 years of age in low- and middle-income countries received antiretroviral therapy (ART) representing 28% of those in need of ART with a substantial variation within African region (12% in Western and Central Africa and 32% in Eastern and Southern Africa) [2].

Data from ongoing randomized clinical trials and observational datasets in Africa have demonstrated a clear mortality benefit from starting ART among children eligible for ART [3-9]. However, in sub-Saharan Africa, the daily management of HIV care for children remains a great challenge for the following reasons: first, diagnosing HIV infection early among children less than 18 months is difficult; second, human resources trained to deliver ART to children are lacking; third, pediatric antiretroviral formulations are limited; fourth, ART is complex in the context of multiple co-morbidities (tuberculosis, malaria, malnutrition, etc.); and lastly, low-income countries suffer from poor health care systems [2,10,11]. In spite of these challenges, data from various pediatric ART programs in Africa report good early clinical outcomes in children [3-5,8]. In adult cohorts, the poor retention, varied between 76-80%, was reported at 12 months in HIV care among patients initiated ART [12-14]. Few reports on retention in pediatric care are available in sub-Saharan Africa.

In July 2006, the National Institute of Health launched the International epidemiological Database to Evaluate AIDS (IeDEA) initiative to better describe the trends of the epidemiology of HIV in the context of ART access in specific regions in the world http://www.iedea-hiv.org In West Africa http://www.iedeawestafrica.org, the IeDEA Pediatric Working Group (pWADA) was established in January 2007 to study the care and treatment of HIV-infected children in this region of sub-Saharan Africa, where the ART coverage among children reported in 2009 was the lowest (12%) [2].

In this context, we aimed to describe the characteristics of HIV-infected children at ART initiation and study the 12-month mortality and loss-to-program rates in the pWADA database.

## Methods

### The IeDEA West Africa collaboration

By collecting and integrating data from multiple HIV/AIDS cohorts, this initiative aims to address unique and evolving research questions in the field of HIV/AIDS care and treatment. In West Africa, this collaboration was initiated in July 2006 and currently involves 10 pediatric HIV/AIDS clinics spread over eight countries. The first merger, done in April 2008, included the data from nine pediatric clinical centers in Benin (n = 1), Côte d'Ivoire (n = 4), Gambia (n = 1), Ghana (n = 1), Mali (n = 1) and Senegal (n = 1).

### Inclusion criteria

In the IeDEA West Africa pediatric HIV center, clinical forms were available for recording HIV activities and an electronic database was available for data entry. We used a standardized data collection procedure to merge the HIV pediatric data [15]. All HIV-1-infected children (positive viral load test < 18 months or positive serology ≥ 18 months) aged < 16 years with documented gender and initiated triple drugs antiretroviral therapy were included irrespective of the first-line ART regimens. The initiation of ART was based on the national or international guidelines [11,16,17].

The following information was recorded in the database: age, gender, body mass index, date of ART initiation, pre-treatment CD4 percentage, cotrimoxazole, initiation and type of ART regimens. HIV-infected children are typically seen in clinics at least every three months, and their CD4 counts are measured twice a year to monitor their immunological response to ART. Routine viral load monitoring is not available in most sites.

### Ethical aspects

The IeDEA West Africa collaboration was approved by the national ethics committees of each country. Individual informed signed consent was waived for this analysis.

### Outcomes

The outcomes of this study were i) death, defined as any death recorded in the database during the first year of follow-up; ii) loss-to-follow-up, indicated when the interval between the last clinic visit registered in the database and the closing date of the database was > 6 months; and iii) loss-to-program, defined as the combined outcome of death or loss-to-follow-up.

The baseline was the date of ART initiation. The explanatory variables included the following: gender, age at ART initiation, 2006 WHO criteria for immunodeficiency according to CD4 cell count or CD4 percent and age [17], cotrimoxazole status at ART initiation, year of ART initiation and first-line antiretroviral regimen (Non-Nucleoside Reverse Transcriptase Inhibitor [NNRTI] or Protease Inhibitor [PI]). The clinical stage of the disease was defined as either less advanced (CDC stage A/B or WHO stage I/II/III/) or advanced (CDC stage C or WHO stage IV).

### Statistical analysis

Continuous variables were compared using the Wilcoxon rank-sum test, and comparisons between two categorical variables were performed using the Chi-square test and Fisher's exact test when appropriate. The Kaplan-Meier method was used to estimate the probability of death or loss to program with their 95% Confidence Interval (CI). The logrank test was used for comparisons between groups. We used Weilbull regression models with random effects to estimate mortality and loss-to-program hazard ratios (HR) with their 95% CI, accounting for heterogeneity between the different programs or cohorts. Models included individual variables (age, sex, pre-ART CD4 count, type of initial regimen and clinical stage of disease, cotrimoxazole initiation and year of ART initiation). The multivariable analysis was performed by the backward selection procedure including all variables (explicative model). The dataset was closed for analysis after the data merger was completed in April 2008. Follow-up was censored at the date of death for children deceased or the date of the last visit for children who were lost to follow-up. Children alive and in care in April 2008 were right-censored at the date of their last visit before this fixed date. All analyses were performed using intent-to-treat approach with SAS software version 9.1 (SAS Institute, Cary, NC, USA).

## Results

### Description of sites

The collaboration included nine pediatric clinical centers from six countries in West Africa (Table 1). Free access to all laboratory tests (CD4 count testing, liver function testing and total blood count) was available in three out of the nine clinical centers (Table 1). All of the children had free access to ART as well as cotrimoxazole prophylaxis expected at the Korbelu Teaching Hospital in Ghana. Four of the clinical centers are located within teaching hospitals. The biggest cohort was located in Bamako, with 673 ART-treated children, and the smallest site was in Gambia, with 23 children enrolled (Table 2).

**Table 1:** Characteristics of the paediatric clinical sites in the IeDEA West African collaboration (pWADA), 2000-2007.

Country	Site	Electronic files or medical records	Location	Sector (public/private)	Free access to ART	Free access to cotrimoxazole	Free access to lab exams*	Availability of tracing methods for loss to follow-up**
**Benin**	Hôpital d'Instruction des Armées, Cotonou (UPEIV)	Electronic	Urban	Private	Yes	Yes	Yes	Yes
**Côte d'Ivoire**	Centre de Prise en charge, de Recherche et de Formation (CEPREF), Abidjan	Electronic	Urban	Public	Yes	Yes	Yes	Yes
**Côte d'Ivoire**	MTCT Plus Network, Abidjan	Electronic	Urban	Public	Yes	Yes	Yes	Yes
**Côte d'Ivoire**	CHU de Yopougon, Abidjan	Electronic	Urban	Public	Yes	Yes	Yes	Yes
**Côte d'Ivoire**	CIRBA	Electronic	Urban	Semi-public	Yes	Yes	Yes	No
**Gambia**	Medical Research Council, Fajara	Medical records	Urban	Public	Yes	Yes	Yes	No
**Ghana**	Korle Bu Hospital, Accra (KBTH)	Medical records	Urban	Public	No	No	Yes	No
**Senegal**	Hôpital d'Enfants Albert-Royer, Dakar	Medical records	Urban	Public	Yes	Yes	Yes	Yes
**Mali**	Hôpital Gabriel Touré, Bamako	Electronic	Urban	Public	Yes	Yes	Yes	No

* Free access to laboratory exams (CD4 count testing, total blood count, liver function testing)

**Tracing methods: used cell phone call or do home visit by social workers team when the patient missed one scheduled visit

**Table 2:** Baseline characteristics at antiretroviral treatment (ART) initiation of 2170 children.

Site	N	Girl (%)	Median Age (years) (IQR)	%AIDS or clinical stage IV (%Missing)	Initial ART regimen (%)			Median CD4 cell count (IQR)	Median CD4 percent (IQR)	%Immuno-supression* (%Missing)	%CTX Status^$ ^(%Missing)	Year of ART initiation		
												
					2NRTI+1NNRTI	2NRTI +1 PI	Others					≤ 2004	2005	≥ 2006
**UPEIV**	71	46.5	4 (2-7)	24.3 (1.4)	0.0	100.0	0.0	291 (132-517)	8(5-12)	89.9 (2.8)	100.0 (1.4)	50.7	19.7	29.6
**CEPREF**	306	42.5	6 (3-9)	20.2 (1.6)	63.1	33.3	3.6	356 (106-728)	11 (6-16)	79.5 (15.4)	32.8 (0.3)	12.7	50.7	36.6
**MTCT Plus**	74	52.7	2 (1-4)	10.3 (21.6)	35.1	64.9	0.0	858 (437-1285)	18 (14-24)	57.7 (4.1)	89.7 (21.6)	41.9	23.0	35.1
**CHU Yopougon**	650	48.3	6(3-10)	0.5 (4.0)	46.0	53.5	0.5	445 (201-738)	14(7-21)	60.1 (14.5)	15.6 (0.6)	52.3	24.2	23.5
**CIRBA**	141	47.5	4 (2-7)	53.3 (5.1)	66.7	31.9	1.4	393 (227-947)	14 (6-20)	72.0 (46.8)	71.2 (48.2)	13.5	19.9	66.7
**FAJARA**	23	47.8	6 (4-12)	- (100.0)	82.6	13.0	4.3	305 (220-580)	15 (9-19)	50.0 (4.3)	- (100.0)	4.3	34.8	60.9
**KBTH**	128	47.7	6 (3-8)	3.9 (0.0)	96.9	0.0	3.1	332 (77-684)	11 (4-17)	65.6 (25.0)	93.8 (0.0)	11.7	51.6	36.7
**Albert Royer**	104	43.3	7 (4-10)	55.3 (1.0)	81.7	15.4	2.9	340 (31-610)	9 (4-15)	63.9 (41.3)	92.3 (0.0)	32.7	20.2	47.1
**Gabriel Toure**	673	40.4	4 (2-8)	30.4 (2.2)	75.2	23.0	1.8	335 (115-602)	-	55.8 (16.3)	- (100.0)	39.8	18.0	42.2

**Total**	**2170**	**44.8**	**5 (2-9)**	**19.1 (13.2)**	**62.0**	**36.3**	**1.7**	**385 (142-691)**	**13 (7-19)**	**63.4 (18.3)**	**42.7 (36.2)**	**36.0**	**27.1**	**36.9**

IeDEA paediatric West Africa database (pWADA), 2000-2007.

IQR: Interquartile range, NRTI: Nucleoside reverse transcriptase inhibitors, NNRTI: Non nucleoside reverses transcriptase inhibitors,

PI: protease inhibitors, KBTH: Korlebu Teaching Hospital, Accra, UPEIV: Hôpital d'Instruction des armées, Cotonou, CTX: Cotrimoxazole

* According to WHO definition [4], $ at ART initiation

### Characteristics of patients and antiretroviral regimens at ART initiation

Between June 2000 and December 2007, 2,170 children were included. The baseline characteristics per center are summarized in table 2. Overall, median age was 5 years (Interquartile range [IQR]: 2-9); median CD4 percentage was 13% (IQR: 7-19); and 51.8% of these HIV-infected children had immunosuppression at ART initiation. All of the sites had similar baseline characteristics except for the MTCT-Plus clinic, which recruited children at a younger median age of two years and a higher median CD4 percentage (18%). Cotrimoxazole was initiated in 27.2% of children (range: 0.0%-98.6%). The first-line treatment combinations were two nucleoside reverse transcriptase inhibitors (NRTI) and one non-nucleoside reverse transcriptase inhibitor (NNRTI) in 62.0% of the cases, followed by two NRTIs and one protease inhibitor (PI) in 36.3% of cases, and the other combinations were used in 1.7% of the cases. The most frequent PI used was Nelfinavir (95.7%); the remaining cases were treated with Lopinavir boosted with Ritonavir.

### Mortality rate and loss-to-program

**Figure 1:**
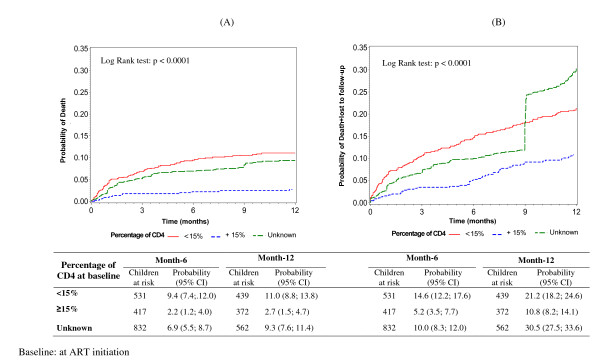
**Kaplan-Meier probability of death (A) or loss to program (death or loss to follow-up) (B) in 2170 children on antiretroviral therapy (ART), by CD4 percentage at ART initiation**. IeDEA pediatric West Africa database (pWADA), 2000-2007.

Among these 2,170 HIV-infected children on ART, 169 (7.8%) died during the follow-up period (1761 child-years) with a mortality rate, estimated to be 9.6 deaths per 100 child-years (95% CI: 9.4-9.7). Overall, the 12-month probability of death after ART initiation was 8.3% (95% CI: 7.2-9.6%), with substantial differences across sites and age categories (p = 0.001) (Table 3). The 12-month probability of death was 11.0% in children with severe immunosuppression (CD4 percentage < 15%) at baseline, 2.7% in those with CD4 percentage ≥ 15% and 9.3% in those with unknown CD4 percentage (p < 0.0001) (Figure 1).

**Table 3:** Probability of death or loss to program in 2170 children on antiretroviral therapy (ART), by age group at ART initiation.

	Death				Loss to follow-up				Loss to program (death and lost to follow-up)			
	Month-6		Month-12		Month-6		Month-12		Month-6		Month-12	
	
Age at ART initiation	n	Probability (95% CI)	n	Probability (95% CI)	n	Probability (95% CI)	n	Probability (95% CI)	n	Probability (95% CI)	n	Probability (95% CI)
**< 12 months**	36	8.9 (3.4; 22.2)	23	11.7 (5.0; 26.0)	36	0.0 (0.0; 0.0)	23	20.2 (10.2; 37.8)	36	8.9 (3.4;.22.2)	23	29.8 (18.0; 46.6)
**1-3 years**	398	10.6 (8.2; 13.7)	278	12.7 (10.0; 16.1)	398	5.0 (3.4; 7.3)	278	19.4 (15.8; 23.7)	398	15.1 (12.2; 18.5)	278	29.7 (25.7; 34.1)
**3-5 years**	341	3.7 (2.2; 6.2)	262	5.2 (3.4; 8.1)	341	2.4 (1.2; 4.6)	262	15.9 (12.3; 20.3)	341	6.0 (4.0; 8.9)	262	20.3 (16.5; 24.9)
**5-10 years**	629	5.6 (4.2; 7.5)	516	7.1 (5.4; 9.2)	629	4.2 (3.0; 6.0)	516	13.8 (11.4; 16.8)	629	9.6 (7.7; 12.0)	516	19.9 (17.1; 23.1)
**≥ 10 years**	376	6.2 (4.3; 8.9)	294	8.1 (5.9; 11.2)	376	4.3 (2.7; 6.7)	294	15.8 (12.5; 19.9)	376	10.2 (7.7; 13.5)	294	22.7 (18.9; 27.0)

IeDEA pediatric West Africa database (pWADA), 2000-2007.

n: number of children at risk

The 12 month probability of lost-to follow-up was 16.0% (95% CI: 14.4-17.8%), and varied according to age group (Table 3).

A total of 461 children were lost-to-program (dead or last visit > 6 months) with an incidence of 26.2 per 100 child-years, 95% CI [25.9-26.4]. The 12-month probability of loss-to-program on ART were 23.1% (95% CI: 21.3-25.0%) overall and varied per age group (p = 0.0005) (Table 3). This probability was 21.2% for a CD4 percentage < 15%, 10.8% for a CD4 percentage ≥ 15% and 30.5% for missing CD4 percentage (p < 0.0001).

### Factors associated with death or loss-to-program

A multivariable analysis was performed adjusting for gender, age, type of ART regimen, clinical stage, CD4 percentage of ART initiation, year of ART initiation, and cotrimoxazole status. The adjusted hazard ratio (aHR) of death was 2.1 (95% CI: 1.1-4.1) for children with CD4 percentage < 15%. It was 1.5 (95% CI: 0.6-3.6) for those with a missing CD4 percentage (reference group was CD4≥ 15% at ART initiation). The aHR was 2.5 (95% CI: 1.6-3.9) for children at an advanced clinical stage at ART initiation. For children who initiated ART in 2005, the aHR was 1.7 (95% CI: 1.0-2.9) compared to those who initiated ART before 2005. The aHR was 2.8 (95% CI: 1.7-4.7) for children who initiated ART in 2006 or after. The remaining variables such as gender, age, first-line ART regimen and cotrimoxazole status were not independently associated with death (Table 4).

**Table 4:** Weibull survival model with random effects in 2131 children on antiretroviral therapy (ART).

	Univariate analysis			Multivariate analysis		
		
	HR*	95% CI	*P*	aHR	95% CI	*P*
**Sex**						
Boys	1	-	-	1	-	-
Girls	1.1	0.7-1.5	0.72	1.0	0.7-1.4	0.88
**Age at ART initiation**						
10 years and plus	1	-	-	1	-	-
[5-10] years	0.8	0.5-1.4	0.49	0.9	0.5-1.5	0.70
[3-5] years	0.6	0.3-1.2	0.15	0.9	0.5-1.7	0.68
[1-3] years	1.5	0.9-2.6	0.12	1.2	0.7-2.1	0.40
< 12 months	1.2	0.4-4.3	0.69	1.1	0.3-3.8	0.79
**ART regimen at baseline**						
2 NRTI+1 NNRTI	1	-	-	1		
2 NRTI+1 PI	0.7	0.4-1.0	0.06	0.9	0.6-1.5	0.76
Other	0.2	0.02-2.6	0.21	1.0	0.2-5.0	0.98
**Clinical stage at ART initiation**						
A, B or I, II, III	1	-	-	1	-	-
AIDS or IV	6.0	3.9-9.2	< 0.01	2.5	1.6-3.9	< 0.01
Unknown	0.9	0.4-2.0	0.90	1.4	0.7-2.9	0.29
**% CD4 at ART initiation**						
≥ 15%	1	-	-	1	-	-
< 15%	2.1	1.1-3.9	0.02	2.1	1.1-4.1	0.03
Missing	1.5	0.7-3.3	0.22	1.5	0.6-3.6	0.27
**Year of ART initiation**						
≤ 2004	1	-	-	1	-	-
2005	1.8	1.0-3.0	0.03	1.7	1.0-2.9	0.04
≥ 2006	2.6	1.7-4.2	< 0.01	2.8	1.7-4.7	< 0.01
**Initiation of cotrimoxazole at ART initiation**						
No	1	-	-	1	-	-
Yes	1.3	0.7-2.5	0.39	0.9	0.5-1.7	0.67
Missing	0.6	0.1-3.1	0.50	0.4	0.1-2.3	0.30

IeDEA paediatric West Africa database (pWADA), 2000-2007. Mortality analysis.

*HR = Hazard Ratio, **95% CI = 95% Confidence Interval, aHR = adjusted Hazard Ratio

Heterogeneity between cohort, p = 0.25

Similar factors were associated with loss-to-program (Table 5): advanced clinical stage (aHR = 2.3, 95% CI (1.7-3.0)); CD4 percentage < 15% (aHR = 1.9, 95% CI (1.3-2.9)); and year of ART initiation. The aHR for children who initiated ART in 2005 was 1.7 (95% CI: 1.3-2.4), and the aHR for those who initiated ART in 2006 and after was 3.0 (95% CI: 2.2-4.0) compared to those who initiated ART before 2005

**Table 5:** Weibull survival model with random effects in 2131 children on antiretroviral therapy (ART).

	Univariate analysis			Multivariate analysis		
		
	HR*	CI (95%)**	*P*	aHR	CI (95%)**	*P*
**Sex**						
Boys	1	-	-	1	-	-
Girls	1.0	0.8-1.3	0.72	1.0	0.8-1.2	0.78
**Age at ART initiation**						
10 years and plus	1	-	-	1	-	-
[5-10] years	0.9	0.6-1.2	0.32	0.9	0.7-1.3	0.56
[3-5] years	0.8	0.5-1.2	0.22	0.9	0.6-1.3	0.52
[1-3] years	1.3	0.9-1.8	0.10	1.1	0.8-1.6	0.34
< 12 months	1.4	0.6-2.9	0.36	1.1	0.5-2.4	0.66
**ART regimen at baseline**						
2 NRTI+1 NNRTI	1	-	-	1		
2 NRTI+1 IP	0.7	0.5-0.9	0.01	0.9	0.7-1.2	0.62
Other	0.6	0.2-1.5	0.24	1.0	0.4-2.5	0.98
**Clinical stage at ART initiation**						
A, B or I, II, III	1	-	-	1	-	-
AIDS or IV	2.6	2.0-3.5	< 0.01	2.3	1.7-3.0	< 0.01
Unknown	1.3	0.8-2.1	0.21	1.4	0.9-2.3	0.15
**% CD4 at ART initiation**						
≥ 15%	1	-	-	1	-	-
< 15%	2.1	1.4.3.2	< 0.01	1.9	1.3-2.9	< 0.01
Missing	1.5	0.9-2.5	0.07	1.7	1.1-2.8	0.03
**Year of ART initiation**						
≤ 2004	1	-	-	1	-	-
2005	2.0	1.5-2.7	< 0.01	1.7	1.3-2.4	< 0.01
≥ 2006	3.5	2.6-4.6	< 0.01	3.0	2.2-4.0	< 0.01
**Cotrimoxazole status at ART initiation**						
No	1	-	-	1	-	-
Yes	1.2	0.7-1.8	0.43	0.9	0.6-1.4	0.63
Missing	0.6	0.2-1.8	0.32	0.4	0.1-1.5	0.16

IeDEA paediatric West Africa database (pWADA), 2000-2007. Loss-to-program analysis.

*HR = Hazard Ratio, **95% CI = 95% Confidence Interval, aHR = adjusted Hazard Ratio

Heterogeneity between cohort, p = 0.14

## Discussion

The IeDEA West Africa collaboration provides an opportunity to collect, analyze and compare access to ART and its field outcomes in this part of the world. After one year on ART, the overall mortality in children was 8.3% and the retention in HIV care programs was 78.8%. The same factors were independently associated with both death and retention in HIV care: an advanced HIV disease, a severe immunosuppression at ART initiation and year of ART initiation.

Similar mortality rates was reported in other studies conducted with HIV-infected children in Africa and, ranging from 6.3% to 11.5% [3,5,7,8,18]. The mortality rate reported in the studied cohorts is likely under-estimated because there was a high rate of loss-to-follow-up in the HIV cohort and lost to follow-up children were severely immune-suppressed, a marker of a higher risk of death. In general, mortality is under-estimated in ART African programs because of the misclassification of LTFU [12,19,20]. An investigation conducted in adult patients LTFU in Malawi found that 50% of them had died, 23% were alive and 27% untraceable [21]. We were not able to classify patients LTFU as their status was not routinely ascertained in the participating centers.

All the participating cohorts reported a late access to ART, except the Abidjan MTCT-Plus group, in which the linkage between PMTCT and HIV postnatal care has been strengthened by early HIV pediatric diagnosis. In the MTCT-Plus group, the median age at ART initiation was 2 years [22]. Indeed, in the current study, only 2% of children initiated ART before their first year age. This observation is consistent with data reported by MSF programs, which reported that only 8.5% of children were less than 12 months at ART initiation [8]. Our findings underline important concerns in the timely identification of HIV-infected children in urban areas of West Africa. The linkage between the PMTCT program and pediatric care is crucial and should be reinforced wherever possible to identify HIV-infected infants earlier [22]. Substantial challenges in pediatric care in low-income countries include identifying HIV-infected children earlier and initiating ART treatment as quickly as possible. The access to an early infant diagnostic for HIV by PCR remains largely uncommon [23,24]. Despite the recent availability of dried blood spots testing, including in rural areas [23-25], the delay between HIV testing and availability of the results remains a concern and requires particular organization. In 2009, only 15% of children born to HIV-positive mothers received an HIV test within the two first two months of life [2].

In the current study, age was not associated with death as reported in the KIDS-ART-LINC Collaboration [3] and also with loss-to-program. We unquestionably enrolled in this observational data the HIV-1 infected long-term non-progressors who survive until 5 years as most of HIV transmission among infants were mother-to-child transmission. Indeed, the mortality in vertically-infected children before the introduction of ART in the first year of life was about 40% and less than 30% in children infected in postnatal period [26].

We reported that 51.8% of children exhibited severe immunosuppression at ART initiation, which was associated with both death and loss-to-program. This association was previously reported for death but not for loss to follow-up [3,27]. The long delay before the diagnostic of pediatric HIV infection in children could also explain the severe immunosuppression observed among HIV-infected children who initiated ART.

HIV-infected children who initiated ART in 2005 or after presented a higher risk of death or loss-to-program compared to children who initiated ART before 2005. Possible explanations for this finding are as follows. First, the children who initiated ART after 2005 were at a more advanced clinical stage and presented more severe immunosuppression than the children who initiated before 2005 (data not shown). Second, there was a substantial increase in the number of HIV-infected children who were followed up in these centers with an increased workload, which may have impacted the standard of care. Indeed, we noted that the number the children who initiated ART between 2005 and 2008 roughly doubled in this period. Third, after 2005, there was an extension of new pediatric clinical centers with the new initiatives and support of US partners. It suggests that existing services are increasingly challenged to cope with the increased load of HIV-infected children due to the rapid scale up of ART programs in West African countries.

In the current study, retention of HIV pediatric care programs was 78.8% in West Africa at 12 months of follow-up. Retention was 85% at 12 months and 75% at 36 months in the pediatric MSF programs [8]. Retention in HIV care is now one of the most important challenges faced by heath care workers and HIV implementing partners as the coverage of HIV care and treatment have improved for both adults and children in low-income countries. Similar findings were reported in adult patients, with a low rate (76%) of retention in HIV care at 12 months in the IeDEA West Africa [12]. Also, a systematic review of adult patients in low-income countries reported that the retention in HIV care at 36 months was 64.6% [13]. Innovative and sustainable approaches to improve retention in HIV pediatric care are needed. For the adult's HIV-infected patients, then keys recommendations was proposed based on the Malawi's' experience [28]. We believe that some of these recommendations could be applied for the pediatric HIV care.

We acknowledge that the present study is not representative of all children on ART in West Africa as most of the data was gathered from urban sites, in which the standard of care may be higher than in rural areas. However, this study allows a comparison of the management of HIV care at the patient level between the programs in six countries in West Africa. In addition, we hypothesize that the operational problems identified here will be worse in less structured clinical care.

One limitation of this study, and generally in large collaborative studies, is the lack of data collected on factors related to access to health care, such as adherence to ART, or social variables, such as orphan status, to better explain mortality in HIV-infected children. These key variables are not routinely collected in the clinical forms used in the different HIV centers. In addition, the analysis did not include nutritional status (weight-for-age, height-for-age) as the data available are not exploitable due to important missing data. Also, data on cotrimoxazole initiation was under-reported, explaining the low rate of children who initiated cotrimoxazole in the collaboration. Another limitation is the quality of data in the HIV cohort in West Africa, specifically in regards to missing data of key variables, such as percentage of CD4 count at baseline (47%). The quality of data collected as well as its validity remains challenging in low incomes countries. However, this merger of data presented a clear picture of the quality of data and provided an example of the challenges of gathering reliable data in the field reality of the HIV pediatric cohorts in West Africa. After the assessment of available data, procedures were put into place in the collaboration to improve the quality of data in the participating centers.

There have been dramatic advances in the daily management of HIV disease in children in West Africa, with overall good clinical outcomes but low retention rates. The IeDEA West Africa collaboration offers the opportunity to increase the quality of data, which remains a concern in sub-Saharan African cohort, as well as to better document clinical outcomes. Comparisons with other IeDEA regions of the world could improve the understanding of the clinical outcomes reported.

## Conclusions

It is urgent to find innovative approaches to early screening of HIV-infected children from six weeks of age onwards to implement large scale and durable pediatric ART programs. It is also important to better document causes of death and loss to program and to propose sustainable approaches to increase retention in HIV pediatric programs in West Africa.

## Competing interests

The authors declare that they have no competing interests.

## Authors' contributions

DKE, AA, KM and VL conceived of the study, and participated in its design and coordination. KM and VL performed the statistical analysis. HS, PT, KK, FET participated at the design of the study and its coordination. DKE, FD, LR, KP and VL drafted the manuscript. All authors read and approved the final manuscript

## Pre-publication history

The pre-publication history for this paper can be accessed here:

http://www.biomedcentral.com/1471-2458/11/519/prepub
